# Sudden cardiac arrest due to coronary vasospasm in a patient with Wolff-Parkinson-White syndrome during brain surgery: a case report

**DOI:** 10.1186/s40981-019-0233-2

**Published:** 2019-02-28

**Authors:** Yuka Matsuki, Maki Mizogami, Kenji Shigemi

**Affiliations:** Department of Anesthesiology and Reanimatology, Faculty of Medicine Sciences, University of Fukui, 23-3 Eiheijicho, Yoshidagun, Fukui, 910-1193 Japan

**Keywords:** Wolff-Parkinson-White syndrome, Coronary vasospasm, Cardiac arrest

## Abstract

**Background:**

Wolff-Parkinson-White (WPW) syndrome has the risk of sudden cardiac death. Without appropriate treatment, coronary vasospasm is also a potentially fatal condition due to ischemia-induced ventricular fibrillation. A rare case of cardiac arrest due to coronary vasospasm during general anesthesia in a patient with pre-existing WPW syndrome is presented.

**Case presentation:**

A 55-year-old man was scheduled for brain surgery under general anesthesia. During surgery, the ECG monitor showed ST segment elevation followed by sustained ventricular tachycardia and the patient’s blood pressure was unmeasurable. Since pseudo-VT with WPW syndrome was suspected, pilsicainide was administered. A few weeks later, a spasm provocation test with acetylcholine was performed, which showed complete spastic occlusion of the right coronary artery.

**Conclusions:**

A rare case of cardiac arrest during surgery in a patient with WPW syndrome, possibly caused by coronary vasospasm, was described.

## Background

Wolff-Parkinson-White (WPW) syndrome is an electrical conduction abnormality in which atrial impulses are transmitted to the ventricle by an accessory pathway, and it presents the risk of sudden cardiac death associated with paroxysmal atrial fibrillation (PAF), so-called pseudo-ventricular tachycardia (pseudo-VT) [1, 2]. Without appropriate treatment, coronary vasospasm is also a potentially fatal condition due to ischemia-induced ventricular fibrillation [3, 4]. A rare case of cardiac arrest due to coronary vasospasm during general anesthesia in a patient with pre-existing WPW syndrome is presented.

## Case presentation

**Fig. 1:**
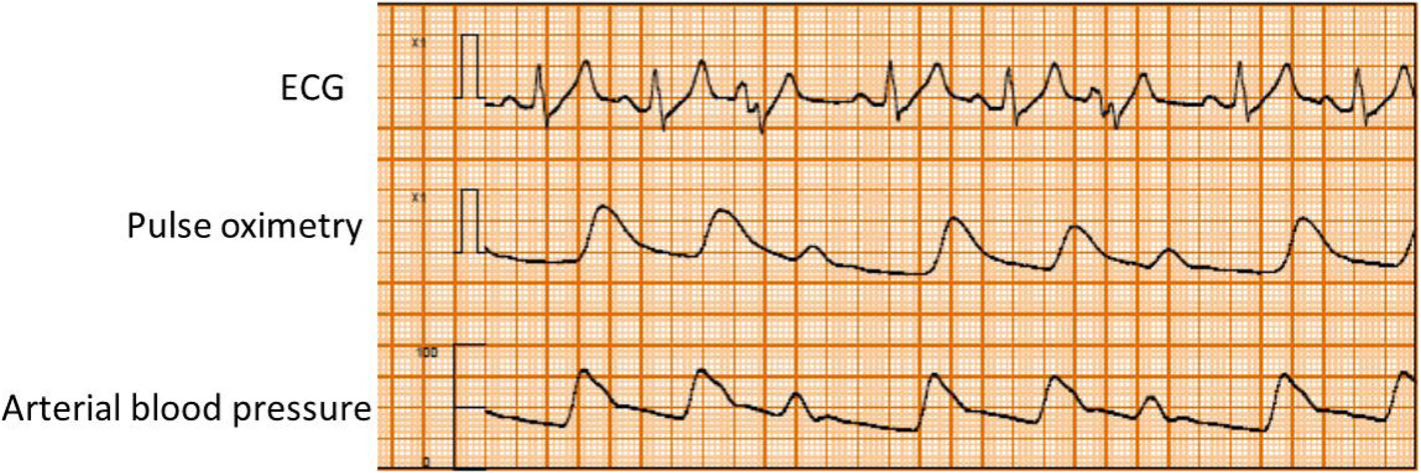
Preoperative electrocardiogram. The tracing of lead II showed complete right bundle branch block and delta waves with WPW syndrome

**Fig. 2:**
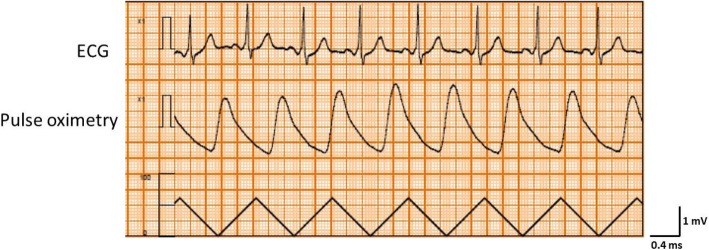
Intraoperative monitor (ECG, pulse oximetry, ABP) after surgery was started. The ECG monitor showed sudden premature ventricular contractions (PVCs) with wide QRS complexes and ventricular bigeminy

**Fig. 3:**
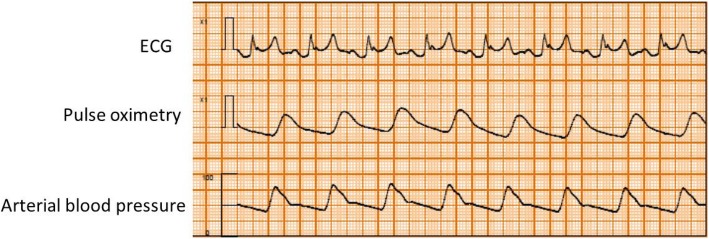
The ECG monitor showed marked ST segment elevation

**Fig. 4:**
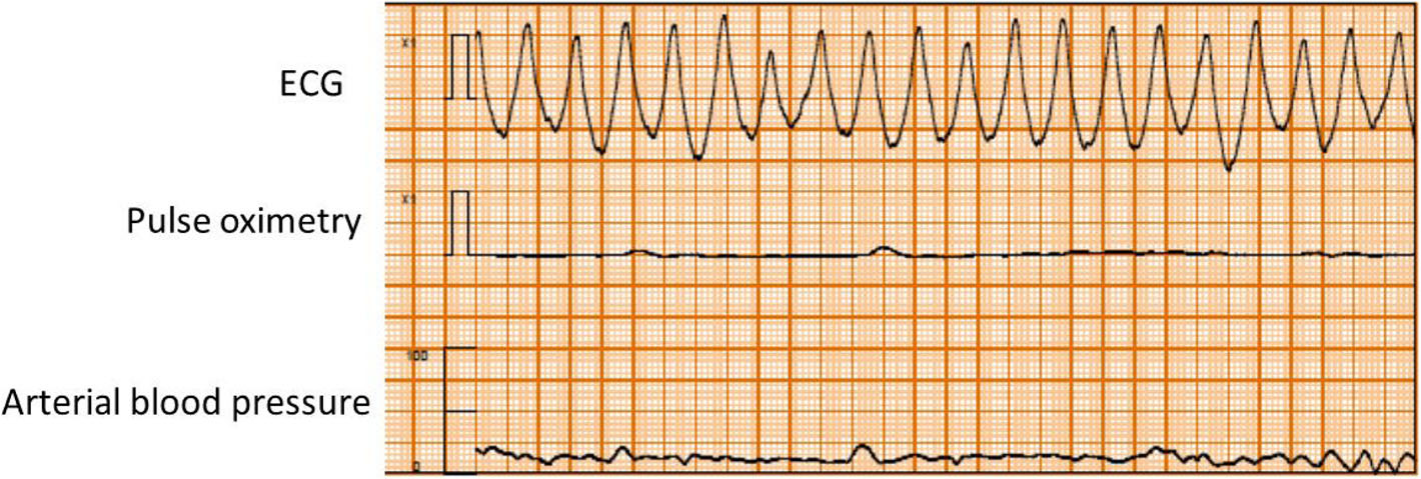
The ECG monitor again showed ST segment elevation followed by sustained ventricular tachycardia. And ABP was unmeasurable

Written, informed consent was obtained from the patient for publication of this case report and accompanying images. A 55-year-old man (height, 170.5 cm; body weight, 66.8 kg; American Society of Anesthesiologists physical status class II) with glioma of the right temporal lobe was scheduled for craniotomy and tumor resection under general anesthesia. He had a history of smoking (Brinkmann Index 300), but no personal or family history of ischemic heart disease. There were no abnormalities in relation to his neurological findings and basic activities of daily life (ADL) before surgery. And no symptoms of intracranial hypertension were found. Preoperative 12-lead electrocardiography (ECG) showed complete right bundle branch block and WPW syndrome with delta waves. Other preoperative examinations including chest X-rays, pulmonary function tests, and blood analyses showed no abnormalities. The patient was taken to the operating room without premedication, where standard monitoring was applied, including ECG leads (focused on lead II) (Fig. 1), non-invasive blood pressure (BP), and pulse oximetry. After peripheral vein cannulation, anesthesia was induced with 0.3 μg/kg/min of remifentanil and 120 mg of propofol. Endotracheal intubation followed after administration of 60 mg of rocuronium. Anesthesia was maintained using 3–5% desflurane in a 40% oxygen/air mixture and 0.1–0.2 μg/kg/min of the remifentanil infusion. The left radial artery was cannulated for invasive arterial blood pressure measurement and collection of blood for analysis. The patient was then placed in the prone position. After rigid head fixation, comprising insertion of three metal pins, surgery was started. About 2 h later, during excision of the cerebral dura mater, the ECG monitor displayed sudden premature ventricular contractions (PVCs) with wide QRS complexes and ventricular bigeminy (Fig. 2). The peripheral blood oxygen saturation (SpO_2_) was 100% and end Tidal CO_2_ (ETCO_2_) was 38 mmHg immediately before the arrhythmia. And bispectral index (BIS) value was 31–38 before the arrhythmia. These were not preceded by changes in heart rate (HR) or arterial blood pressure (HR 80 beats min, arterial blood pressure (ABP) 94/47 mmHg) and disappeared completely after intravenous administration of 50 mg lidocaine while surgery was temporarily interrupted. However, as soon as surgery resumed, the ECG monitor showed marked ST segment elevation lasting about 2 min (HR 80 beats min, ABP 82/45 mmHg) (Fig. 3). Intravenous nicorandil (2 mg/h) was administered, and the procedure abandoned after considering the perioperative risk factors, such as possible severe underlying cardiac pathology and the risk of surgery in the prone position. The serum potassium level of arterial blood gas analysis was normal (4.1 mmoL/L, normal range 3.5–5.0 mmoL/L). Approximately 13 min later, during closure of the dura mater, the ECG monitor again showed ST segment elevation followed by sustained ventricular tachycardia (200 beats per minute; Fig. 4). The patient’s blood pressure was unmeasurable shortly afterwards. Though cardioversion was once thought to be hard to perform in the prone position, the entire episode lasted about 1 min and resolved spontaneously. Since pseudo-VT with WPW syndrome was suspected, a continuous infusion of pilsicainide, a Class I(c) antiarrhythmic, was started at 22 mg/kg/min. Once surgery concluded the patient was immediately turned from the prone to the supine position and transferred to the intensive care unit. The patient had an uneventful recovery, with normal postoperative 12-lead ECG and cardiac enzyme measurements (including creatine kinase and lactate dehydrogenase). Two weeks after surgery, a spasm provocation test with acetylcholine was performed, which showed complete spastic occlusion of the right coronary artery, and this confirmed the possible diagnosis of the intraoperative ST elevation due to coronary vasospasm. Postoperatively, the patient agreed to further evaluation of his intraoperative symptoms. While an electrophysiological study showed the presence of a posterolateral accessory pathway, consistent with the preoperative diagnosis of WPW syndrome, the anterograde effective refractory period (ERP) of the abnormal accessory pathway was noted to be relatively long (540 ms). The patient underwent successful catheter ablation using radiofrequency current, and his posttreatment ECG showed resolution of the inferior Q waves. Three months later, the patient underwent successful reoperation using preoperative antiplatelet therapy and perioperative nicorandil infusion.

## Discussion

A case of cardiac arrest due to coronary vasospasm during neurosurgical anesthesia in a patient with WPW syndrome was described. WPW syndrome is thought to affect 0.13–0.30% of the general population [5, 6], and its characteristic ECG shows a short PR interval, a wide QRS complex, and a delta wave [7, 8]. This cardiac conduction disorder may not always be detected preoperatively, because it is rarely recognized without an accompanying arrhythmia. In the present patient, the diagnosis was still not confirmed, but assumed to be WPW pattern by the presence of delta waves in the 12 leads. A key problem in the anesthetic management of these patients is the risk of tachyarrhythmias such as PAF or pseudo-VT [ 1,2]. Because the atrial excitation bypasses the AV node and travels to the ventricle through an accessory pathway (the bundle of Kent) with a short refractory period, the QRS complex is thus widened by the delta wave, causing a VT-like waveform. About 10–32% of patients with WPW syndrome can develop a life-threatening tachyarrhythmia [1, 2]. In WPW syndrome, electrophysiological studies are often used to identify high-risk patients prone to a rapid ventricular response during atrial fibrillation. As the ERP of the accessory tract is usually longer than that of the normal AV nodal His-Purkinje tract, patients with a short ERP carry higher probabilities of developing symptoms or complications. In this case, the pseudo-VT pattern in WPW syndrome was initially suspected, but judged to be unlikely due to a sufficiently long ERP of 540 ms in the accessory pathway shown in the electrophysiological study conducted 3 weeks postoperatively. Coronary angiography with intracoronary spasm provocation testing is the only certain and effective method for the definite diagnosis of significant coronary artery spasm. Coronary vasospasm is common, even among patients without a prior history of angina, and surgery or anesthesia itself may be strong triggers for vasospasm [3]. Coronary vasospasm should always be considered as a possible cause of cardiac arrest. In this case, it may have been triggered by intraoperative hypotension or vagal stimulation possibly due to dural traction. In patients with coexistent WPW syndrome and ischemic heart disease, coronary vasospasm may produce more profound myocardial ischemia during surgical management [9]. This is complicated by the fact that it is difficult to interpret ischemic ECG abnormalities using only a standard intraoperative ECG monitor in the perioperative period. ECG monitoring is the only reliable practical method to diagnose vasospastic angina during general anesthesia, with 97% of patients showing ST elevation. Of these, approximately 20% will develop ventricular fibrillation or cardiac arrest [3, 4]. One of the confusing ECG findings in both myocardial ischemia and WPW syndrome is the presence of Q waves: a pathologic Q wave and an initial deflection of the delta wave for myocardial ischemia and WPW, respectively [10]. ST segment changes may also confound diagnosis. Tamagna et al. concluded that only ischemic areas large enough to produce ST segment changes were detectable underlying the WPW pattern. Smaller areas, producing only T wave changes, would not induce changes resulting in reliable diagnosis of myocardial ischemia with WPW patterns [11]. This latter situation likely applied in the present case.

A rare case of cardiac arrest during brain surgery in a patient with WPW syndrome, possibly caused by coronary vasospasm, was described. Although it was extremely difficult to diagnose intraoperatively, appropriate and early treatment led to a good outcome. Anesthesiologists should be aware that ECG changes caused by WPW syndrome may be confused with ischemic ones, which may result in life-threatening complications during general anesthesia.

## Abbreviations

ABP: Arterial blood pressure
BP: Blood pressure
ECG: Electrocardiography
ERP: Effective refractory period
HR: Heart rate
PAF: Paroxysmal atrial fibrillation
PVCs: Premature ventricular contractions
VT: Ventricular tachycardia
WPW: Wolff-Parkinson-White
